# Chimeric antigen receptor T cells in the fast lane among autoimmune disease therapies

**DOI:** 10.1002/cti2.1502

**Published:** 2024-04-12

**Authors:** Zhoujie Ding, David Tarlinton

**Affiliations:** ^1^ Department of Immunology Monash University Melbourne VIC Australia

## Abstract

In this commentary, we highlight recent studies demonstrating the feasibility and promise of chimeric antigen receptor (CAR) T‐cell therapy in treating a number of autoimmune disorders including systemic lupus erythematosus and compare CAR T cells to other therapies aimed at depleting B‐lineage cells in treating such diseases.

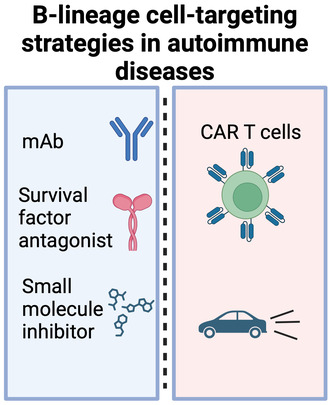

The first chimeric antigen receptor (CAR) T‐cell therapy was approved by the U.S. Food and Drug Administration (FDA) in 2017 for treating acute lymphoblastic leukaemia. Now this strategy to target and eliminate pathogenic cells is being applied beyond cancer to treat other life‐threatening diseases including autoimmune diseases and with spectacular preliminary results. While this raises the possibility of CAR T‐cell therapy being approved for treating autoimmune diseases such as systemic lupus erythematosus (SLE) in the future, care will be needed in its application as those diseases are highly heterogenous and the therapy is not without risk.

Autoimmune disorders are characterised by the ongoing production of auto‐antibodies (Abs) causing inflammation and organ damage. As such, autoreactive B cells and their descendant Ab‐secreting cells (ASCs) are central both to the pathogenesis of many autoimmune diseases and to multiple treatment strategies developed over many years. The success of anti‐CD19 CAR T cells in treating certain B‐cell cancers led to a pioneering study in 2021 whereby autologous anti‐CD19 CAR T cells were used to treat one patient with severe, refractory SLE.^1^ Remarkably, that patient achieved both serologic and clinical remission within 7 weeks of a single CAR T‐cell infusion.^1^ The same research groups followed up their initial study with another in 2022 involving five patients with refractory SLE. Again, all entered remission within 3 months of the autologous anti‐CD19 CAR T‐cell infusion,^2^ which was maintained drug‐free with a median follow‐up of 8 months, even after the reappearance of B cells.^2^ In a very recently reported study, anti‐CD19 CAR T‐cell therapy had great efficacy in 15 patients with either severe SLE (eight patients), idiopathic inflammatory myositis (three patients) or systemic sclerosis (four patients).^3^ Of note, SLE‐specific auto‐Ab levels dropped below the detection cut‐off for almost all the autoantigens tested in all eight SLE patients at the 6‐month follow‐up and remained so during the up to 2‐year follow‐up period.^3^ These most recent SLE patients, as in the earlier studies,^1^, ^2^ all achieved drug‐free remission within 6 months of the CAR T‐cell infusion and remained so for up to 2 years.^3^ Collectively, these studies demonstrate the feasibility and remarkable promise of CAR T‐cell therapy in treating SLE and a broadening span of autoimmune disorders.

Prior to CAR T cells, several therapies aimed at depleting B cells and/or ASCs have been explored in autoimmune diseases with varying success^4^: (1) monoclonal Abs (mAbs) specific for B‐lineage surface proteins, (2) mAbs or soluble receptors blocking essential survival factors or (3) small molecules inhibiting critical biochemical processes (Figure 1). While these treatments very likely have effects beyond depleting B‐lineage cells, in this commentary, we are focusing on comparing their B lineage‐targeting effects with CAR T‐cell therapy on autoimmune diseases. Rituximab (anti‐CD20 mAb), for example, is very effective in treating some auto‐Ab‐associated diseases, reflected in its FDA approval for anti‐neutrophil cytoplasmic auto‐Ab (ANCA)‐associated vasculitis and pemphigus vulgaris (PV), where in both cases the vast majority of patients achieved short‐term, complete remission in contrast to controls.^5^, ^6^ Although small‐scale trials suggested that rituximab could be effective in SLE, large‐scale, phase III clinical studies revealed only minor impacts^7^, ^8^; a 30–40% reduction in auto‐Ab titres relative to pre‐treatment baseline amounts and very modest improvement in disease symptoms over the placebo group, despite > 90% of the patients achieving complete peripheral B‐cell depletion.^7^, ^8^ While the overall ineffectiveness may in part be because of the mode of action or tissue penetration of the mAb, the striking difference in outcome for rituximab in different diseases must mean that the origin or aetiology of the pathogenic auto‐Abs is different. Simplistically, CD20 expression is downregulated on all mature ASCs^9^ so they would escape rituximab‐mediated depletion, whereas CD20‐positive plasmablasts and immature ASCs would remain sensitive. Hence, auto‐Abs coming from different ASCs would predict varying sensitivity to rituximab. But this also raises interesting questions: Why would targeting CD19 be more effective than CD20 in certain autoimmune diseases, given their reasonably similar distribution across B‐cell lineage (Figure 1)? And, if Ab is the driver, shouldn't ASC‐focused therapies be effective? Is the difference related to heterogeneity among ASCs?

**Figure 1:**
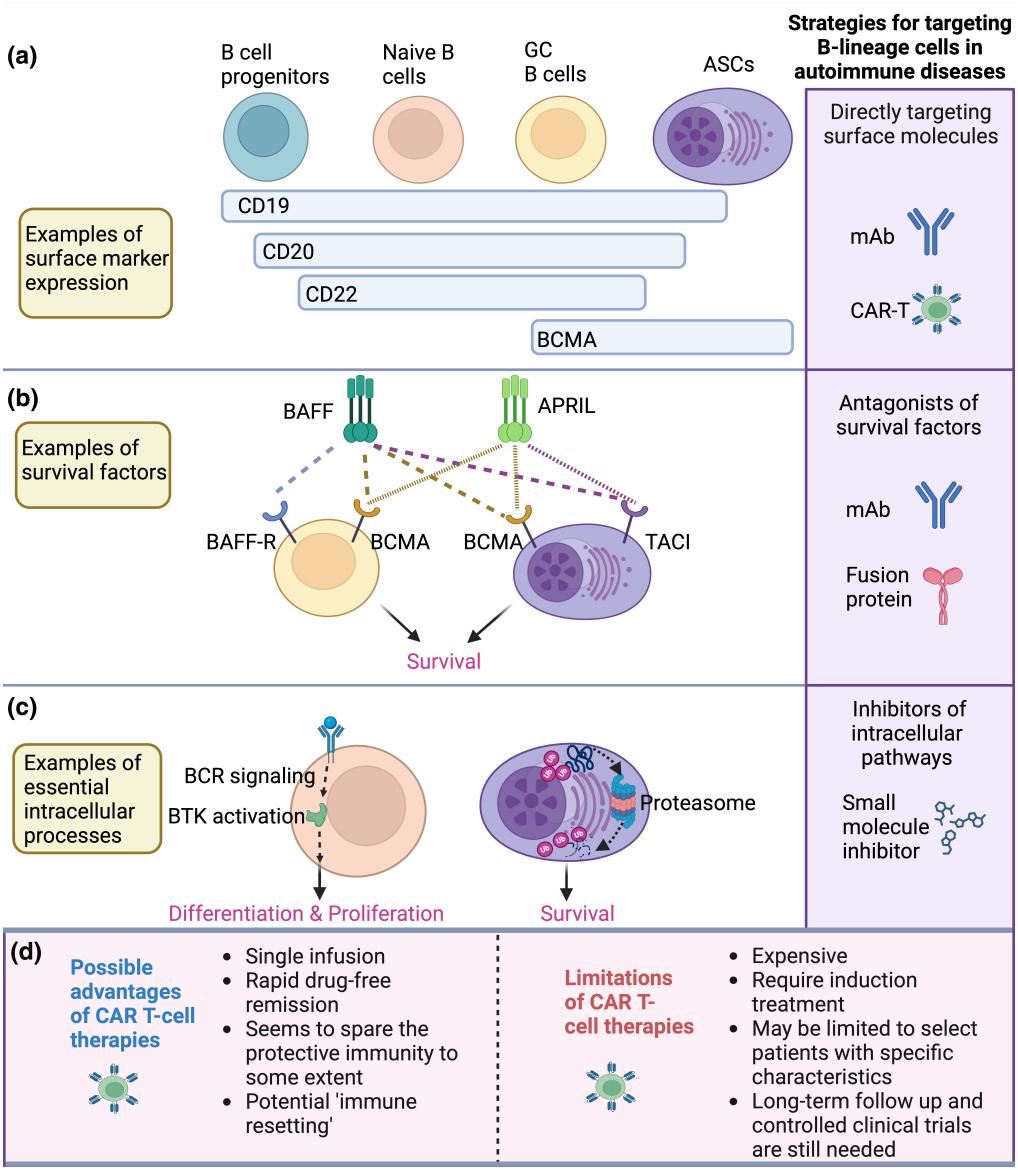
Therapeutic strategies that target B‐lineage cells in autoimmune diseases. **(a)** B‐lineage cells can be targeted directly via mAbs or CAR‐T cells that recognise specific surface markers on the B‐lineage cells. **(b)** B cells and ASCs can be suppressed by mAbs or recombinant proteins antagonising survival factors such as BAFF and APRIL. **(c)** B cells and ASCs can be suppressed by small molecule inhibitors targeting essential intracellular components such as the Bruton's tyrosine kinase (BTK) and the proteosome. **(d)** Advantages and limitations of CAR‐T‐cell therapies compared to the other B‐lineage‐targeting strategies. BCMA, B‐cell maturation antigen; GC, germinal centre. This figure was created with BioRender.

There is compelling evidence that targeting ASCs can be an effective treatment for SLE. Long‐term treatment of SLE patients in a randomised phase II/III clinical trial with atacicept, a transmembrane activator and calcium modulator and cyclophilin ligand interactor (TACI)‐Ig Fc fusion protein that targets survival of B cells and ASCs by blocking binding of B‐cell activating factor (BAFF) and a proliferation‐inducing ligand (APRIL) to their receptors, lead to sustained reductions of both total Abs and auto‐Abs.^10^ The high‐dose arm of the trial was halted prematurely after two unexpected deaths linked to severe respiratory infections but patients who completed that treatment prior to discontinuation showed positive clinical responses.^10^ Such an outcome has underpinned reassessment of the efficacy and safety of TACI‐Ig Fc fusion protein and, indeed, telitacicept, a novel variant of the TACI‐Ig Fc fusion protein, has received initial approval in China for treating active SLE.^11^ The proteasome inhibitor bortezomib (Btz), approved for multiple myeloma, efficiently targets ASCs and has been trialled several times in refractory SLE (examples in Walhelm *et al*.^12^). While some studies reported successes, others were more circumspect and noted serious adverse events indicating that use of Btz will likely remain highly selective and require careful monitoring. But the positive clinical experience of Btz, atacicept—and recently, daratumumab (anti‐CD38 mAb)^13^—highlight the potential of targeting ASCs, certainly in a subset of patients. Interestingly, the two biologicals that are approved for treatment of SLE, belimumab,^14^ a BAFF antagonistic mAb‐targeting B‐cell survival, and anifrolumab,^15^ an interferon α/β receptor subunit 1 (IFNAR1)‐targeting mAb that dampens inflammation, both significantly reduce symptoms in moderate to severe SLE, but neither has drug‐free remission as a common outcome.^14^, ^15^ So, on a superficial level, CAR T treatments may appear superior to current options, but the data are from phase III trials and extensive real‐world use versus pilot studies in restricted patient groups. It remains crucial to determine the extent to which the current outcomes with CAR T therapies are reproducible in SLE patients more generally and to balance those benefits against the risks and costs.

If anti‐CD19 CAR T‐cell therapy does indeed turn out to be more efficient and/or effective than existing treatments in even a subset of autoimmune disorders, a speculation based on extremely restricted data,^3^ it raises the question of why that might be so. It is puzzling given the overlap in target expression of anti‐CD19 CAR T cells with rituximab and atacicept. The difference may be technical, with CAR T cells being better at killing B‐lineage cells throughout the whole body including the bone marrow,^16^ resulting in more thorough B‐lineage cell depletion than that achieved by rituximab, belimumab or atacicept. Alternatively, it may be that by eliminating the earliest B‐cell precursors together with CD19‐positive ASCs, anti‐CD19 CAR T‐cell therapy ‘resets’ the patients' immune environment to ‘normal’, creating an extended symptom and treatment‐free period. Or, it could reflect that pathogenic ASCs in SLE are overwhelmingly CD19‐positive, meaning that they are killed by CAR T cells and not replaced as a result of B‐cell precursor loss. However, if anti‐CD19 CAR T treatment outcomes differ from atacicept, it would suggest these long‐lived, pathogenic ASCs are independent of APRIL.

It is worth noting that in the fifteen patients with autoimmune diseases receiving anti‐CD19 CAR‐T cells, their serum levels of vaccine‐induced Abs against measles, mumps, and rubella decreased only moderately during the 2‐year follow‐up and they contracted only mild infections that were resolved by antiviral or antibiotic treatments,^3^ indicating maintenance of immune memory. This is consistent with both childhood vaccine specificities being enriched in the CD19‐negative bone marrow ASC population,^9^, ^17^, ^18^ and with CD19‐negative ASCs being present in bone marrow aspiration samples from B‐cell cancer patients post anti‐CD19 CAR‐T‐cell infusion who also retained certain vaccine/pathogen reactivities.^19^ Somewhat confusingly, however, vaccine‐specific Ab levels were also largely retained in atacicept‐treated patients,^10^ indicating either those vaccine‐specific ASCs reside in a CD19‐negative, APRIL‐independent compartment or there is a broad distribution of specificities among bone marrow ASC compartments, which is suggested by other studies.^20^, ^21^ The complete characterisation of the different subpopulations of B cells and ASCs in both health and autoimmune disease will greatly assist in improved targeting of pathogenic, autoreactive B cells and ASCs.

The hope for CAR T‐cell therapies in autoimmune disorders is reflected also in the number of clinical trials initiated in the last 7 years. As of March 2024, there were over 30 clinical trials registered at clinicaltrials.gov for CAR‐T and CAAR‐T‐cell treatments spanning 20 autoimmune indications. Currently, CAR T‐cell therapy requires stringent patient selection as they must first stop receiving immunosuppressive drugs and go through lymphodepleting chemotherapy before the CAR T‐cell infusion, during which disease symptoms may dramatically worsen. While it has been suggested that SLE patients infused with anti‐CD19 CAR T cells may achieve ‘immune reset’,^3^ long‐term follow‐up and larger, controlled clinical trials are required to fully assess efficacy. Moreover, while anti‐CD19 CAR T‐cell therapies have shown promising outcomes in treating some autoimmune disorders, these diseases are highly heterogenous. That is, a more complete characterisation of the autoreactive B cells and ASCs and the mechanisms of how they contribute to the different autoimmune disorders will be important in revealing how to precisely match the therapy to the disease, killing the pathogenic cells while leaving protective immunity intact. This would be a revolution in treatment.

## Conflict of interest

The authors declare no conflict of interest.

## Author contributions

**Zhoujie Ding:** Conceptualization; writing – original draft; writing – review and editing. **David Tarlinton:** Conceptualization; writing – review and editing.
